# Antibiotic dispensing during the COVID-19 pandemic: analysis of Welsh primary care dispensing data

**DOI:** 10.1093/fampra/cmab141

**Published:** 2021-11-10

**Authors:** Diana R Wasag, Rebecca Cannings-John, Kathryn Hughes, Haroon Ahmed

**Affiliations:** 1 Division of Population Medicine, School of Medicine, Cardiff University, Cardiff, United Kingdom; 2 Centre for Trials Research, College of Biomedical & Life Sciences, School of Medicine, Cardiff University, Cardiff, United Kingdom

**Keywords:** antibiotic dispensing/prescribing, COVID-19, general practice, RTIs, UTIs

## Abstract

**Background:**

The COVID-19 pandemic led to rapid changes in demand and delivery of primary care services that could have led to increases in antibiotic prescribing.

**Objective:**

We investigated the impact of the COVID-19 pandemic on oral antibiotic dispensing rates in primary care in Wales using longitudinal analysis of monthly oral antibiotic dispensing data from 1 April 2018 to 30 April 2021.

**Methods:**

We used All-Wales primary care dispensing data. We examined trends in oral antibiotic dispensing per 1,000 people for Wales and for individual Health Boards. We used interrupted time series analysis to estimate changes in trends pre- and post-April 2020 to assess the impact of the first and subsequent lockdowns.

**Results:**

Between April 2020 and April 2021, antibiotic dispensing in Wales was lower compared with the same period in 2018 and 2019, with an average monthly decrease of 14.00 dispensed items per 1,000 registered patients (95% confidence interval 19.89–8.11). The overall prepandemic monthly antibiotic dispensing rate ranged from 48.5 to 67.4 antibiotic items per 1,000 registered patients. From the onset of the pandemic, it ranged from 40.3 to 49.07 antibiotic items per 1,000 registered patients. This reduction was primarily driven by narrow-spectrum antibiotics. Statistically significant reductions were also observed for antibiotics commonly dispensed for the treatment of respiratory tract infections. Dispensing of antibiotics primarily used for urinary and skin infections remained stable.

**Conclusions:**

Despite complexities of consulting during the COVID-19 pandemic in primary care we found no evidence of an increase in antibiotic dispensing during this time.

**Key Messages**

- Antibiotic dispensing from onset of the COVID-19 pandemic has decreased.
- This decrease was driven mainly by narrow-spectrum antibiotics.
- Dispensing rates of broad-spectrum antibiotics remained stable.
- Dispensing of antibiotics primarily used for urinary and skin infections remained stable.

## Introduction

Most antibiotic prescribing occurs in the community. In 2019, 71% of antibiotics dispensed in the NHS in England were prescribed by Primary Care.^1^ An estimated 9%–23% of antibiotics prescribed in English Primary Care between 2013 and 2015 were potentially clinically unnecessary.^2^ Widespread prescribing of antibiotics is thought to increase antimicrobial resistance (AMR), which poses a serious challenge to patient safety and provision of health services in the years to come.^3^ Substantial progress has been made in recent years to reduce rates of antibiotic prescribing in response to concerns over rising rates of AMR. The observed reduction in the total number of antibiotic items dispensed in England from 2015 to 2019 was 14%,^1^ and in Wales from 2013 to 2018 was 11.9%.^4^

The COVID-19 pandemic led to accelerated adoption of remote consulting in UK primary care.^5^ In England, the number of telephone appointments in general practice increased by 270% between April and August 2020 in comparison to the corresponding period in 2019.^6^ Considerable numbers of people with respiratory tract symptoms were triaged remotely, there was uncertainty around COVID-19 illness, and some antibiotics (e.g. Azithromycin) were promoted as potentially beneficial.^7^ These issues overlap with key factors known to influence prescribing such as clinical uncertainty and workload time pressures.^8^ Furthermore, previous studies found that antibiotics are frequently overprescribed for respiratory tract infections (RTIs),^9^ and influenza like illness accounts for substantial inappropriate antibiotic use.^10^ Therefore, many clinicians were concerned that antibiotic dispensing would rise during the pandemic due to remote consulting, increased clinical uncertainty, and higher prevalence of respiratory symptoms in the community.

Despite initial concerns, recent studies of antibiotic prescribing trends primarily observed reductions in use. In the Netherlands prescribing of antibiotics decreased during the COVID-19 pandemic from 600 per 100,000 patients to 360 per 100,000 patients, especially for RTIs.^11^

In the United Kingdom as a whole, antibiotic prescribing fell below predicted rates as compared with the previous year between April and August (antibiotic prescribing adjusted rate ratio [ARR] ranging from 0.73 to 0.91), but higher rates were observed in March 2020 (ARR = 1.13) compared with other months between February and September 2020.^12^ In Scotland, an initial 44% increase in the number of prescriptions for commonly used antibiotics for RTIs was observed in the week ending 22 March 2020, compared with the corresponding week in 2019.^13^ However, by the end of May 2020, 34% fewer prescriptions were issued compared with the corresponding week in 2019.^13^ In North-West London, from March to November 2020, a decrease of 3,504 antibiotic items per month was observed (compared with decrease of only 584 items per month prior to the onset of pandemic).^14^

In contrast, the United States observed that 72% of COVID-19 patients received antibiotics, even when there was no evidence of secondary bacterial infection.^15^ A large meta-analysis of 154 empirical studies concluded that 3 quarters of patients with COVID-19 received antibiotics, while bacterial coinfection was estimated to be present in only 8.6% of cases.^16^

Wales traditionally has high rates of antibiotic prescriptions.^17^ As per the Welsh Government Antibacterial Stewardship Plan 2019, prepandemic there has been a decrease in the total antibiotic use among almost all Health Boards in Wales with a significant reduction in prescriptions of agents used for the treatment of respiratory infections such as amoxicillin and macrolides.^4^ Therefore, due to higher rates of antibiotic prescribing compared with the rest of the United Kingdom, previously published data from the United Kingdom as a whole,^12^ Scotland,^13^ or London^14^ might not be representative of rates of antibiotic dispensing in Wales during the pandemic.

Most previous research reporting antibiotic usage during the COVID-19 pandemic used prescribing rates. However, between 11% and 19% of prescriptions are not dispensed,^18^ and thus dispensing rates are likely more reflective of actual usage than prescribing rates. While generally reflecting prescribing practices, dispensing rates provide better insight into antibiotic usage in the population. Therefore, in this research we evaluated whether antibiotic usage had changed in Wales during the COVID-19 pandemic using All-Wales dispensing data. We hypothesized that increased rates of remote consulting and respiratory symptoms may have led to an increase in antibiotic dispensing following the onset of the COVID-19 pandemic.

## Methods

### Study population and data source

This was a longitudinal analysis of oral antibiotic dispensing data in Wales from 1 April 2018 to 30 April 2021.

In Wales, General Practitioners (GPs) provide free of charge universal healthcare. GPs work within specific geographical areas called Health Boards, and each Health Board provides antibiotic guidance based on local patterns of AMR which assists clinicians when making decisions about antibiotic prescribing.

General Practice Prescribing Data (CASPA) is maintained by the NHS Wales Shared Services Partnership. It includes information on all antibiotics prescribed in Wales by GPs and nonmedical prescribers in each practice. This information is available as practice-level aggregated data and no clinical or demographic data are available about individual patients, or details of consultation or diagnosis. For the prescription to be included in the dataset, it must be dispensed in the community within Wales or England (having been prescribed in Wales). Prescription data included antibiotic details such as antibiotic name, route of administration, British National Formulary code, and month it was dispensed, as well as details of individual unique GP practice code, and their associated Health Board.

Dispensing was used rather than prescribing in this study. Dispensing is not the same as prescribing as some patients may not take their prescriptions to a pharmacy for dispensing. However, dispensing can be argued to be a better marker of clinical necessity as in some circumstances delayed prescriptions might be used, or patients may choose not to collect their antibiotics, with dispensing data reflecting actual use better than prescribing data.

### Antibiotic dispensing data

We extracted numbers of antibiotic items dispensed in general practices in Wales per month between April 2018 and April 2021. The first day of lockdown in Wales was the 23rd of March 2020. Dispensing data were available on a monthly, not daily basis and therefore, for the purpose of the interrupted time series (ITS) analysis, April 2020 was treated as the start of lockdown.

Antibiotic items were combined to allow for antibiotic dispensing trends to be examined at the All Wales and Health Board levels. We initially examined the number of items dispensed per month for all antibiotics in Wales, and then for commonly prescribed antibiotics for RTIs and ear, nose, throat (ENT) conditions (including amoxicillin, doxycycline, clarithromycin, erythromycin, phenoxymethylpenicillin, and co-amoxiclav) and commonly prescribed antibiotics for urinary tract infections (such as trimethoprim, nitrofurantoin, fosfomycin and pivmecillinam); as well as flucloxacillin, often used for treatment of skin infections (based on British National Formulary coding) (Supplementary Table 1). These antibiotics are referred to later in this paper as antibiotics used for RTI and ENT conditions, urinary tract infections, and skin infections. These are the first line antibiotics recommended by most recent 2018 NICE guidance for treatment of these conditions. The only exception is co-amoxiclav which is recommended as second line for some respiratory conditions and can be used for treatment of multiple other infections. The data were limited to oral preparations only including both tablets and suspensions. Broad-spectrum antibiotics were classified as per the UK 5-year AMR plan.^19^ Broad-spectrum antibiotics included co-amoxiclav, cephalosporines, and quinolones. All other antibiotics have been classified as narrow spectrum for the purpose of this analysis.

### Antibiotic dispensing rates

The numerator used to calculate antibiotic dispensing rates was the number of antibiotic items dispensed. The denominator was an estimate of the population of Wales overall and in each Health Board area. Denominator estimates were calculated based on numbers of patients registered at each General Practice each month. There were small fluctuations in monthly counts due to patients changing practices or due to temporary patient registrations (such as students). The All-Wales monthly denominator counts in our analysis ranged from 3,176,644 to 3,242,279 per month, similar to the mid-2019 Welsh population count of 3,152,879.^20^ Dispensing rates were expressed as number of antibiotic items dispensed per 1,000 registered patients.

### Statistical analysis

Monthly dispensing rates and 95% confidence intervals (CIs) were calculated at all Wales and Health Board level. Dispensing rates over time were displayed graphically to compare the prepandemic period (April 2018–March 2020) to the pandemic period (April 2020–April 2021). Dispensing trends were analysed for all Wales, by individual Health Boards and by commonly used antibiotics. An ITS approach compared dispensing trend prepandemic to the pandemic. This analysis was performed using ordinary-least squares regression with Newey–West standard errors and a lag for the autocorrelation structure. The Cumby–Huizinga test for autocorrelation was examined to determine the appropriate autocorrelation structure to be accounted for in the model. The model included prepandemic and during pandemic trends, as well as a coefficient to examine a step change in level immediately postpandemic (March–April 2020). The parameter estimates are presented alongside 95% CIs and *P* values. Analyses were performed using the *itsa* command in Stata V.16.^21^

## Results

We identified 6,270,703 antibiotic items dispensed between April 2018 and April 2021. Monthly estimates for the whole Welsh population ranged from 39.3 antibiotic items per 1,000 registered patients (in February 2021) to 67.4 antibiotic items per 1,000 registered patients (in December 2019). The overall antibiotic dispensing rate was 54.9 antibiotic items per 1,000 patients at the start of study period (April 2018) and 42.4 antibiotic items per 1,000 patients at the end (April 2021) (Supplementary Table 2). The overall prepandemic (from April 2018 to March 2020) antibiotic dispensing rate in general practice in Wales ranged monthly from 48.5 to 67.4 antibiotic items per 1,000 patients. From the onset of the pandemic (April 2020–April 2021), it ranged from 39.3 to 49.1 antibiotic items per 1,000 patients. Overall, we found similar levels of antibiotic dispensing in 2018 to corresponding months in 2019. However, during the COVID pandemic (from April 2020 until April 2021) lower rates of antibiotic dispensing were seen in corresponding months (Supplementary Tables 2 and 3).

**Fig. 1.**
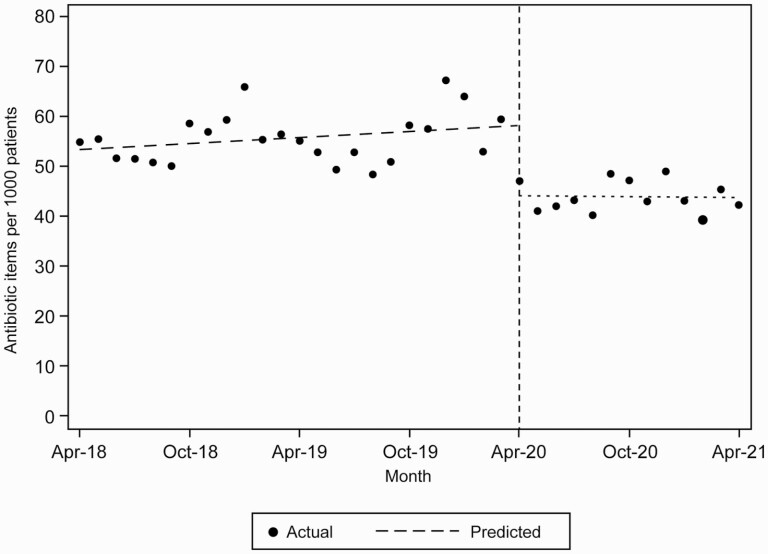
Predicted and actual antibiotic dispensing rates per 1,000 registered patients between April 2018 and April 2021.

The ITS analysis found a statistically significant decrease in dispensing rates (14.00 items per 1,000 registered patients, 95% CI: 19.89–8.11) from April 2020 to April 2021 in comparison to predictions based on dispensed number of antibiotics between April 2018 and March 2020 (Fig. 1 and Supplementary Table 4).

**Fig. 2.**
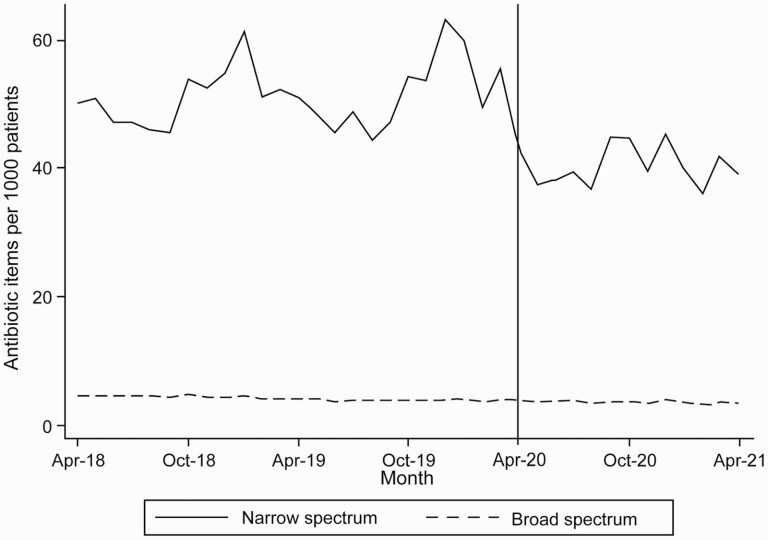
Monthly number of broad- and narrow-spectrum antibiotics dispensed in Wales per 1,000 registered patients between April 2018 and April 2021.

This reduction in overall antibiotic dispensing was primarily driven by fewer narrow-spectrum antibiotics (Fig. 2). Average dispensing rates for each quarter of the year are reported (Supplementary Table 3). The ITS analysis found a statistically significant decrease in narrow-spectrum antibiotics dispensing rates (14.05 items per 1,000 registered patients, 95% CI: 19.88–8.21) from April 2020 to April 2021 in comparison to predictions based on number of dispensed antibiotics between April 2018 and March 2020. A small, nonstatistically significant increase in broad-spectrum antibiotic dispensing was observed for the same time period of 0.16 items per 1,000 registered patients (95% CI: −0.01 to 0.32).

**Fig. 3.**
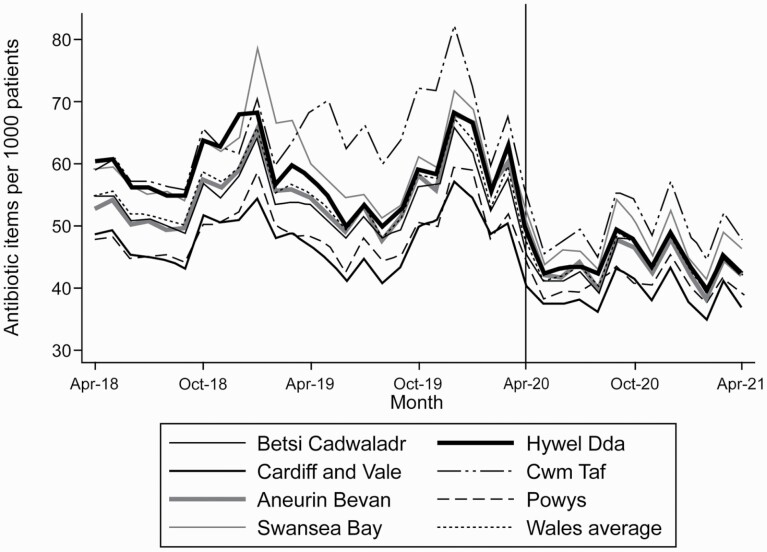
Rates of antibiotic dispensing per 1,000 registered patients across Welsh Health Boards between April 2018 and April 2021.

In March 2020 there was a peak in antibiotic dispensing in Wales of 59.5 items per 1,000 patients compared with 56.6 in March 2019 (Fig. 3 and Supplementary Table 2). This increase in dispensing was observed across all Welsh Health Boards. Monthly rates of antibiotic dispensing in 2020 were only higher in January 2020 than in March 2020. There is a clear variability between Health Boards in the rate of dispensed antibiotics; Health Boards associated with higher rates of dispensing remained high dispensers during the pandemic.

**Fig. 4.**
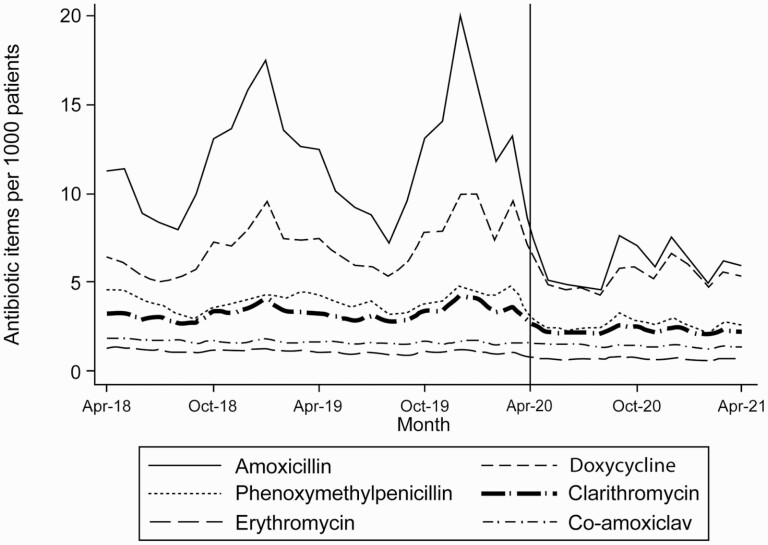
Monthly number of antibiotic dispensing for RTIs and common ENT problems between April 2018 and April 2021.

Amongst the reduced number of dispensed antibiotics, the number of items of antibiotics commonly used for treatment of respiratory and ENT conditions changed markedly (Table 1 and Fig. 4). The data are presented here for summer months (April–July) to avoid issues with seasonal variability. The rates of dispensing of items commonly used for the treatment of urinary tract and skin infections remained relatively stable during the pandemic.

**Table 1.** Comparison of mean rates of dispensed antibiotic items in Wales during the COVID-19 pandemic (April–July 2020) and previous years (April–July 2018 and April–July 2019).

	April 2018–July 2018	April 2019–July 2019	April 2020–July2020	Change between April–July 2019 and April–July 2020	95% CI		*P* value
	Items per 1,000 patients	Items per 1,000 patients	Items per 1,000 patients	Items per 1,000 patients			
Amoxycyllin	9.97	10.18	5.63	4.55	1.91	7.18	0.006
Doxycycline	5.74	6.48	5.22	1.26	−0.19	2.70	0.078
Clarithromycin	3.11	3.08	2.32	0.77	0.41	1.13	0.002
Erythromycin	1.27	1.05	0.72	0.34	0.25	0.42	0.000
Phenoxymethylpenicillin	4.23	3.93	2.56	1.37	0.88	1.85	0.001
Co-amoxiclav	1.79	1.62	1.55	0.07	−0.04	0.18	0.180
Trimethoprim	5.77	4.29	3.70	0.59	0.32	0.85	0.002
Nitrofurantoin	4.58	5.57	5.71	0.14	−0.66	0.38	0.547
Fosfomycin	0.04	0.31	0.49	0.18	−0.27	−0.10	0.002
Pivmecillinam	0.10	0.28	0.40	0.12	−0.18	−0.05	0.004
Flucloxacillin	7.53	7.07	6.55	0.52	−0.89	1.92	0.402

## Discussion

### Summary of key findings

This study assessed the impact of the COVID-19 pandemic on the number of oral antibiotic items dispensed in Wales. Following an initial increase in March 2020, we observed a marked reduction in the number of items dispensed, especially in those used for the treatment of RTIs. For amoxicillin and doxycycline, the number of antibiotic prescriptions dispensed in general practice between April and July 2020 was 140,799 (33.5%) lower than the figure for the corresponding period in 2019 (211,698). Rates of broad-spectrum antibiotic dispensing remained steady during the pandemic.

There was a reduction in antibiotic dispensing from April 2020 to May 2020 (in May 2020 easing of restrictions started in England). Between May and August 2020 much lower antibiotic dispensing rates were observed in comparison to 2019. From August to September 2020, there was a subsequent recovery in rates of antibiotic dispensing. This might reflect easing of COVID-19 restrictions in Wales from mid-July to September 2020. In October and November 2020, further restrictions were reintroduced, which corresponded again to lower rates of antibiotic dispensing. From December 2020 to March 2021, much lower antibiotic dispensing rates were observed in comparison to 2019. This may have been due to the introduction of another national lockdown in the United Kingdom between December 2020 and March 2021.

### Comparison with existing literature

The overall number of antibiotic items dispensed in 2019 of 673 items per 1,000 patients was similar to recent estimates from England (607 items per 1,000 in 2015).^22^ Our overall dispensing rates are lower than reported by Public Health Wales for previous years.^4^ This may be because we only included oral preparations whereas Public Health Wales analyses included topical, parental, inhaled, rectal, and genital, as well as oral preparations.^4^

Prescribing and dispensing have decreased in recent years with the aim of reducing unnecessary antibiotic prescribing in the United Kingdom. The most recent data from Wales show a 7% reduction in dispensed total antimicrobial volume in the 2018/2019 financial year compared with 2017/2018,^17^ with an 11.9% reduction over the 5-year period from 2013 to 2018.^4^ This reduction is similar to the reported 12.2% decrease in total antibiotic consumption in England between 2015 and 2019.^1^ Data from The Netherlands also showed a decline in the number of antibiotics prescribed since the onset of pandemic, particularly for RTIs.^11^ However, data from the United States reported increased usage of antibiotics during the pandemic.^15^ Data from North-West London suggested a decrease in the number of antibiotics prescribed from March to November 2020, in comparison to the same period in 2019.^14^ In England, the number of antibiotic prescriptions made in general practice between April and August 2020 was 15.48% lower than in the corresponding period in 2019, but the number of antibiotic prescriptions was 6.71% higher than expected when accounting for the decrease in the absolute number of GP appointments during this period.^6^ In Scotland, an initial increase was observed from the third week of March in the number of prescriptions issued for the treatment of RTIs, followed by a decrease below prepandemic levels towards the end of May 2020.^13^ A population-based primary care cohort study across all nations of the United Kingdom has described similar findings with antibiotic prescribing declining below predicted rates between April and August 2020.^12^

### Strengths and limitations

This study is unique, unlike previously published research from the United Kingdom as a whole,^12^ Scotland,^13^ and North-West London,^14^ as it uses dispensing rates of antibiotics rather than prescribing rates. There are pros and cons to using both prescribing rates and dispensing rates of antibiotics. It could be argued that prescription rates might more accurately reflect how clinical practice has adapted to the pandemic, as they represent the clinical decision making process, which excludes patient behaviour such as medication adherence. However, clinical decision cannot always be easily measured as clinicians sometimes use delayed prescriptions which are prescribed but not necessarily intended to be used. Between 11% and 19% of prescriptions for any type of medications are not dispensed to patients. Antibiotics are the most commonly prescribed category of nondispensed drug.^23^ In antibiotic stewardship terms, antibiotic dispensing is more important than antibiotic prescribing. Data from Canada comparing antibiotic prescribing and dispensing suggested that primary care electronic medical records should not be used to monitor population level antibiotic use over time. These records are more appropriate to study differences in antibiotic prescribing at physician level than population-based trends.^24^ We therefore believe that dispensing rates provide better insight into antibiotic usage in the population than prescribing rates.

Unfortunately, the data were only available as number of antibiotics issued by each GP practice in Wales, and we were not able to access details of individual consultations. Therefore, we were unable to obtain information about indication for antibiotic prescription, or patients sociodemographic characteristics. Only General Practice level data were available for this analysis. Dispensing data from other locations such as out-of-hours services, community resource teams, incontinence services, and community pharmacists not directly employed by General Practices are not included. No clinical information was available regarding the indication for antibiotics as we had no access to individual clinical records. We did not have access to the overall number of consultations in Wales during the period of the pandemic. Therefore, further studies looking at prescribing within consultation rates would provide useful insight into how antibiotic prescribing might have changed in General Practice due to different workloads.

### Implications for clinical practice

The COVID-19 pandemic appears to have had a substantial impact on dispensing of antibiotics at the population level in Wales, with significant reduction in antibiotics used for RTIs. This decline could be due to reduced primary care attendance or as social distancing measures were in place, due to lower rates of self-limiting viral RTIs in the community. Significant differences in dispensing rates were demonstrated between different Health Boards and recognizing this variability should help policymakers and practitioners to mitigate any inconsistency in antibiotic prescribing long term.

## Conclusion

Our findings suggest that antibiotic stewardship priorities have not been overlooked during the pandemic. The rates of dispensing of broad-spectrum antibiotics remained largely stable. Moreover, steady rates of dispensing for nonrespiratory conditions such as skin and urinary tract infections suggest that the public were still seeking medical advice for some common infections. Patients might have been more reluctant to consult GPs for common RTIs and/or there may have been lower rates of self-limiting viral illness and flu in the community, compared with previous years due to social distancing measurements. Further research is needed to evaluate the possible reasons for the reductions in antibiotic prescribing and dispensing during the Pandemic, such as looking more closely at the content of GP consultations and any changes to patients’ health-seeking behaviour.

## Funding

No founding was required for this study.

## Ethical approval

No ethical approval was necessary as data are widely available in public domain.

## Conflict of interest

None declared.

## Data availability

The data used in this research are freely available at NHS Wales Shared Services Partnership: https://nwssp.nhs.wales/ourservices/primary-care-services/general-information/data-and-publications/general-practice-prescribing-data-extract/.

## Supplementary Material

cmab141_suppl_Supplementary_Material

cmab141_suppl_Supplementary_Checklist
