# Supplementary material for: Antibiotic dispensing during the COVID-19 pandemic: analysis of Welsh primary care dispensing data
Source: Fam Pract. 2021 Nov 10;39(3):420–5. doi: 10.1093/fampra/cmab141 (PMC8822399; doi:10.1093/fampra/cmab141)
Supplement: cmab141_suppl_Supplementary_Material [file cmab141_suppl_supplementary_material.docx]

**Supplementary material**

**Table 1 NICE first line recommended antibiotics for Respiratory Tract (RT) and Ear Nose Throat (ENT) Infections, lower Urinary Tract Infections(UTIs) and skin infections (NICE, 2018)**

| RT and ENT infections | UTIs | Skin infections |
| --- | --- | --- |
| Amoxicillin  Doxycycline  Clarithromycin  Erythromycin  Phenoxymethylpenicillin | Trimethoprim  Nitrofurantoin  Fosfomycin  Pivmecillinam | Flucloxacillin |

Reference: : <https://www.nice.org.uk/Media/Default/About/what-we-do/NICE-guidance/antimicrobial%20guidance/summary-antimicrobial-prescribing-guidance.pdf>

**Table 2 Number of antibiotics prescribed per 1000 patients across all Welsh Health Boards and on average in Wales during each month from April 2018 until April 202**

**Table 3 Average antibiotic dispensing rates per quarter from April 2018 to April 2021**

| Months | Quarter | 2018 | 2019 | 2020 | 2021 |
| --- | --- | --- | --- | --- | --- |
| January-March | 1 |  | 59.29 | 58.89 | 42.68 |
| April-June | 2 | 54.10 | 52.51 | 43.46 | 42.41 |
| July-September | 3 | 50.92 | 50.79 | 44..08 |  |
| October-December | 4 | 58.37 | 61.07 | 46.47 |  |

**Table 4 Parameter estimates from the interrupted time series analysis examining the change in level and slope of dispensed antibiotic prescriptions in primary care following the start of pandemic in the UK in April 2020 (n=37 months)**

|  | **coefficient** | **95% confidence interval** | **p-value** |
| --- | --- | --- | --- |
| intercept | 53.45 | 50.04 to 56.85 | <0.001 |
| pre-pandemic slope | 0.19 | -0.10 to 0.50 | 0.187 |
| during pandemic slope | -0.03 | -0.39 to 0.33 | 0.851 |
| pre-pandemic vs pandemic | -0.23 | -0.71 to 0.24 | 0.328 |
| change in level (step change) | -14.00 | -19.89 to -8.11 | <0.001 |
