# Supplementary material for: Antibiotic dispensing during the COVID-19 pandemic: analysis of Welsh primary care dispensing data
Source: Fam Pract. 2021 Nov 10;39(3):420–5. doi: 10.1093/fampra/cmab141 (PMC8822399; doi:10.1093/fampra/cmab141)
Supplement: cmab141_suppl_Supplementary_Checklist [file cmab141_suppl_supplementary_checklist.docx]

STROBE Statement—Checklist of items that should be included in reports of ***cross-sectional studies***

|  | Item No | Recommendation | Page No |
| --- | --- | --- | --- |
| **Title and abstract** | 1 | (*a*) Indicate the study’s design with a commonly used term in the title or the abstract | Pg 2 longitudinal analysis and time series stated in objective and methods |
|  |  | (*b*) Provide in the abstract an informative and balanced summary of what was done and what was found | Pg 2 |
| Introduction | | | |
| Background/rationale | 2 | Explain the scientific background and rationale for the investigation being reported | Pg 3-5 |
| Objectives | 3 | State specific objectives, including any prespecified hypotheses | Pg 5 : dispensing might be better measurement than prescribing |
| Methods | | | |
| Study design | 4 | Present key elements of study design early in the paper | Pg 5-8 |
| Setting | 5 | Describe the setting, locations, and relevant dates, including periods of recruitment, exposure, follow-up, and data collection | Pg 5-8 |
| Participants | 6 | (*a*) Give the eligibility criteria, and the sources and methods of selection of participants | Pg 5-8 |
| Variables | 7 | Clearly define all outcomes, exposures, predictors, potential confounders, and effect modifiers. Give diagnostic criteria, if applicable | Pg 5-8 |
| Data sources/ measurement | 8* | For each variable of interest, give sources of data and details of methods of assessment (measurement). Describe comparability of assessment methods if there is more than one group | *Pg 5-8* |
| Bias | 9 | Describe any efforts to address potential sources of bias | Adjusted for monthly changes in population size Pg .8 No selection bias as all prescriptions dispensed in Wales included -Pg 6-8 |
| Study size | 10 | Explain how the study size was arrived at | Pg 8-for population size, Pg 8 for number of antibiotic dispensed |
| Quantitative variables | 11 | Explain how quantitative variables were handled in the analyses. If applicable, describe which groupings were chosen and why | Pg 6 and 7 |
| Statistical methods | 12 | (*a*) Describe all statistical methods, including those used to control for confounding | Pg 7 and 8 |
|  |  | (*b*) Describe any methods used to examine subgroups and interactions | Looked at data at all Wales level but also by individual Health Boards and separately for commonly used antibiotics-Pg 8 |
|  |  | (*c*) Explain how missing data were addressed | We looked at all prescriptions dispensed in Wales. The only prescriptions not included were when script was issued but prescription wasn’t taken for dispensing Pg 13 |
|  |  | (*d*) If applicable, describe analytical methods taking account of sampling strategy | N/A as no sampling was implimated, looked at whole data |
|  |  | (*e*) Describe any sensitivity analyses |  |
| Results | | | |
| Participants | 13* | (a) Report numbers of individuals at each stage of study—eg numbers potentially eligible, examined for eligibility, confirmed eligible, included in the study, completing follow-up, and analysed | Pg 6& 7 |
|  |  | (b) Give reasons for non-participation at each stage | Not appropiate |
|  |  | (c) Consider use of a flow diagram | Not appropriate |
| Descriptive data | 14* | (a) Give characteristics of study participants (eg demographic, clinical, social) and information on exposures and potential confounders | Such information not available, as includes all prescriptions dispensed in Wales and whole Welsh population -Pg 6&7 |
|  |  | (b) Indicate number of participants with missing data for each variable of interest | Pg 6 and 7 |
| Outcome data | 15* | Report numbers of outcome events or summary measures | Pg 9-10 |
| Main results | 16 | (*a*) Give unadjusted estimates and, if applicable, confounder-adjusted estimates and their precision (eg, 95% confidence interval). Make clear which confounders were adjusted for and why they were included | Pg 9-10 |
|  |  | (*b*) Report category boundaries when continuous variables were categorized | Pg 10, table 1, figure 4- describes commonly used antibiotics for respiratory, ENT, skin and urinary problems |
|  |  | (*c*) If relevant, consider translating estimates of relative risk into absolute risk for a meaningful time period | Not relevant |
| Other analyses | 17 | Report other analyses done—eg analyses of subgroups and interactions, and sensitivity analyses | Pg 8 The Cumby-Huizinga test for autocorrelation |
| Discussion | | | |
| Key results | 18 | Summarise key results with reference to study objectives | Pg 10-11 Summary of key findings |
| Limitations | 19 | Discuss limitations of the study, taking into account sources of potential bias or imprecision. Discuss both direction and magnitude of any potential bias | Pg 12-strength and limitation section |
| Interpretation | 20 | Give a cautious overall interpretation of results considering objectives, limitations, multiplicity of analyses, results from similar studies, and other relevant evidence | Pg 10 and 11-comaprison with existing literature |
| Generalisability | 21 | Discuss the generalisability (external validity) of the study results | Pg 11-12 |
| Other information | | | |
| Funding | 22 | Give the source of funding and the role of the funders for the present study and, if applicable, for the original study on which the present article is based | Pg 14 |

*Give information separately for exposed and unexposed groups.

**Note:** An Explanation and Elaboration article discusses each checklist item and gives methodological background and published examples of transparent reporting. The STROBE checklist is best used in conjunction with this article (freely available on the Web sites of PLoS Medicine at http://www.plosmedicine.org/, Annals of Internal Medicine at http://www.annals.org/, and Epidemiology at http://www.epidem.com/). Information on the STROBE Initiative is available at www.strobe-statement.org.
